# Beneficial effects of reading aloud and solving simple arithmetic calculations (learning therapy) on a wide range of cognitive functions in the healthy elderly: study protocol for a randomized controlled trial

**DOI:** 10.1186/1745-6215-13-32

**Published:** 2012-04-06

**Authors:** Rui Nouchi, Yasuyuki Taki, Hikaru Takeuchi, Hiroshi Hashizume, Takayuki Nozawa, Atsushi Sekiguchi, Haruka Nouchi, Ryuta Kawashima

**Affiliations:** 1 Smart Ageing International Research Centre, Institute of Development, Aging and Cancer, Tohoku University, Sendai 980-8575, Japan; 2 Japanese Society for the Promotion of Science, Tokyo 102-8472, Japan; 3 Division of Developmental Cognitive Neuroscience, Institute of Development, Aging and Cancer, Tohoku University, Sendai 980-8575, Japan; 4 Department of Functional Brain Imaging, Institute of Development, Aging and Cancer, Tohoku University, Sendai 980-8575, Japan

## Abstract

**Background:**

Almost all cognitive functions decline with age. Results of previous studies have shown that cognitive training related to everyday life (reading aloud and solving simple arithmetic calculations), namely learning therapy, can improve two cognitive function (executive functions and processing speed) in elderly people. However, it remains unclear whether learning therapy engenders improvement of various cognitive functions or not. We investigate the impact of learning therapy on various cognitive functions (executive functions, episodic memory, short-term memory, working memory, attention, reading ability, and processing speed) in healthy older adults.

**Methods:**

We use a single-blinded intervention with two parallel groups (a learning therapy group and a waiting list control group). Testers are blind to the study hypothesis and the group membership of participants. Through an advertisement in local newspaper, 64 healthy older adults are recruited. They will be assigned randomly to a learning therapy group or a waiting list control group. In the learning therapy group, participants are required to perform two cognitive tasks for 6 months: reading Japanese aloud and solving simple calculations. The waiting list group does not participate in the intervention. The primary outcome measure is the Stroop test score: a measure of executive function. Secondary outcome measures are assessments including the following: verbal fluency task, logical memory, first and second names, digit span forward, digit span backward, Japanese reading test, digit cancellation task, digit symbol coding, and symbol search. We assess these outcome measures before and after the intervention.

**Discussion:**

This report is the first study which investigates the beneficial effects of learning therapy on a wide range of cognitive functions of elderly people. Our study provides sufficient evidence of learning therapy effectiveness. Most cognitive functions, which are correlated strongly with daily life activities, decrease with age. These study results can elucidate effects of cognitive training on elderly people.

**Trial registration:**

This trial was registered in The University Hospital Medical Information Network Clinical Trials Registry (No. UMIN000006998).

## Background

Cognitive function changes during a person's lifetime [1]. Elderly people might experience a decline in several cognitive functions such as memory [2], attention [3], executive functions [4,5], and processing speed [6]. Decline in cognitive ability engenders difficulty in performing basic daily living activities [7-9]. Consequently, maintaining or improving cognitive function in older adults is drawing increasing attention [10-25].

Although human cognitive function typically declines with age, earlier studies showed that several cognitive training programs can improve cognitive functions such as memory [18,26], processing speed [21,27,28], executive function [29,30], and attention [31] in healthy elderly people. It is particularly interesting that some studies have demonstrated that the effects of cognitive training can impact non-trained cognitive functions or tasks [30,32-35]. For instance, Schmiedek [35] conducted a working memory training study for elderly people in which participants were required to perform auditory recognition, discrimination, and memory tasks for about 15 min per day at least 5 days per week, for 8 to 10 weeks. Elderly people in the training group showed improvement of cognitive function in directly trained tasks (for example, alpha span and word list) and in cognitive functions in non-trained tasks (for example, animal span, rotation span, word pairs). Results of earlier studies show that cognitive training (for example working memory training) can improve cognitive functions in elderly people.

In line with previous studies using cognitive training for elderly people, we recently developed a new mode of cognitive training using reading aloud and solving of simple arithmetic calculations, namely learning therapy [30,36]. Learning therapy is designed for stimulation of the frontal cortex (especially dorsolateral prefrontal cortex) and of the temporal and parietal association cortices by cognitive tasks, thereby engendering improvement of the function of these cortices [36]. We specifically targeted these regions for the following reasons: (1) Previous functional magnetic resonance imaging (MRI) studies showed that task-related activation of these regions in older adults is lower than that in younger adults [37-42]; (2) Previous structural MRI studies using voxel-based morphometry (VBM) showed that regional gray and white matter volumes of these regions decline with age [43-45]; and (3) These activity and regional gray matter volumes of these regions are closely linked to cognitive functions [39,46-52] such as executive functions, processing speed, and memory, which decrease with age. Therefore, cognitive decline in elderly people might result from reductions of activities and volume in these regions. Based on these facts, we assumed that stimulation of the frontal cortex (especially the dorsolateral prefrontal cortex), as well as those of the temporal and parietal association cortices by cognitive tasks might improve activities and regional gray matter volumes of these cortices. Moreover, they might engender improvement of the functions of these cortices [10,11,18,36,53].

Learning therapy used two simple and easy training tasks (reading Japanese aloud and solving simple arithmetic calculations) derived from knowledge of neuroscience. Results of brain imaging studies indicate that reading sentences or words aloud [54-58] and simple arithmetic operations [59-61] activate the three associated cortices, especially the prefrontal cortex. Reading aloud is accomplished using a combination of several cognitive processes such as recognition of visually presented words, conversion to phonological representation from graphic representation of words, analysis of the meaning of words, and control of pronunciation. Solving arithmetic problems is also accomplished through the use of numerous cognitive processes such as recognition of visually presented numbers, arithmetic operations, and control of hand movements. Moreover, the bilateral prefrontal cortices are activated even when solving very simple and easy problems. Both reading aloud and solving arithmetic problems require working memory. This prefrontal stimulation might engender the positive transfer effect on other cognitive functions. Learning therapy has outstanding features compared to previous cognitive training. First, training tasks of learning therapy are based on results of neuroscience. Secondly, the training tasks are extremely simple and easy for elderly people to perform. Consequently, elderly people can readily comprehend and perform training tasks.

Previous studies using learning therapy have demonstrated that learning therapy can improve executive functions and processing speed in healthy elderly people. For instance, Uchida and Kawashima [30] conducted a randomized controlled trial using learning therapy for healthy elderly people. Participants were divided into learning therapy and control groups. The learning therapy group was required to do two training tasks for 5 days a week: reading Japanese aloud and conducting simple calculations. After 6 months, the learning therapy group showed improved scores in the frontal assessment battery (FAB at bedside), which measures executive function [62-64], and a digit-symbol substitution test, which measures processing speed [65]. These results suggest that learning therapy beneficially affects some cognitive functions in elderly people.

### Purpose of this study

An earlier study showed effects of learning therapy transferred to executive functions and processing speed [30]. However, it remains unclear whether or not the effects of learning therapy can transfer (improve) other cognitive functions such as memory and attention in elderly people. Consequently, the purpose of this study is to investigate whether or not learning therapy can transfer to a wide range of cognitive functions in elderly people. To reveal transfer effects of learning therapy on cognitive functions, we conduct a single-blinded randomized control trial using learning therapy. Testers are blinded to the study hypothesis and the group membership of participants. To evaluate the transfer effects of the reading aloud and solving simple arithmetic calculations interventions (learning therapy), we assess a broad range of cognitive functions. The measured cognitive functions are divisible into seven categories: executive functions, episodic memory, short-term memory, working memory, reading ability, attention, and processing speed.

## Method

### Randomized controlled trial design and setting of this trial

This study, which was registered in the University Hospital Medical Information Network (UMIN) Clinical Trial Registry (UMIN000006998), is a randomized controlled trial conducted in Sendai city, Miyagi prefecture, Japan. Written informed consent to participate in the study will be obtained from each participant before enrolment. The protocol of this study and informed consent were approved by the Ethics Committee of the Tohoku University Graduate School of Medicine.

**Figure 1:**
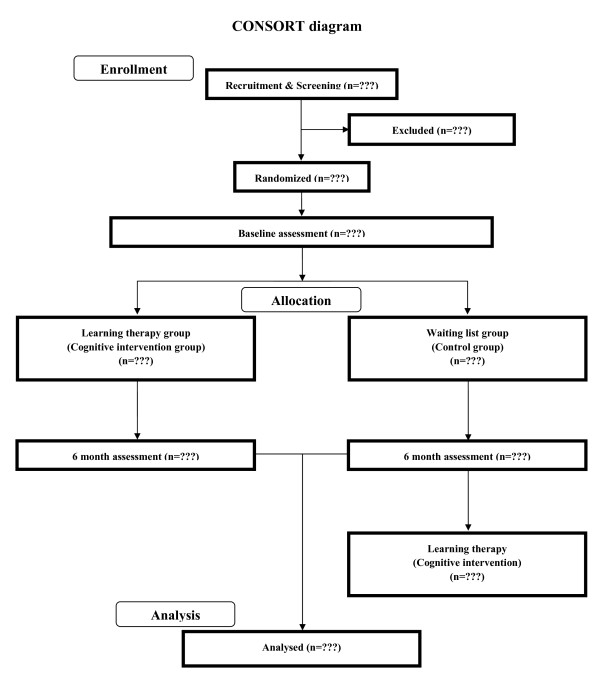
**CONSORT flowchart**.

To assess the impact of learning therapy on a wide range of cognitive functions in healthy elderly people, we use a single-blinded intervention with two parallel groups: a learning therapy group and a waiting list control group. Testers are blind to the study's hypothesis and the group membership of participants. The Consolidated Standards of Reporting Trials (CONSORT) statement [66]http://www.consort-statement.org/home/ has been used as a framework for developing the study methodology (Additional file 1). The trial design is shown in Figure 1.

### Recruitment and selection of participants

Participants are recruited from the general population through advertisements in the local town paper and local newspaper. Interested participants are screened using a semi-structured telephone interview. After the telephone interview, participants are invited to visit Tohoku University for a more detailed screening assessment and to provide written informed consent.

### Inclusion and exclusion criteria

The purpose of this intervention is to investigate transfer effects of learning therapy for a range of cognitive functions in healthy older adults. The criteria include participants who report themselves to be right-handed, native Japanese speakers, unconcerned about their own memory functions, not using medications known to interfere with cognitive functions (including benzodiazepines, antidepressants or other central nervous agents), and having no disease known to affect the central nervous system, including thyroid disease, multiple sclerosis, Parkinson disease, stroke, severe hypertension (systolic blood pressure is over 180, diastolic blood pressure is over 110), and diabetes. Age of participants is over 65 years old. Criteria exclude participants who have an Intelligence Quotient (IQ) less than 85 derived from Japanese Reading Test (JART) [67]. Participants who will participate in another cognitive-related intervention studies will be excluded.

### Randomization

Randomization is designed to take place after receiving the informed consent statement. A researcher (RN) with no contact with the study participants randomly assigns participants to either the learning therapy or the waiting list group by random draw using an online computer program http://www.graphpad.com/quickcalcs/index.cfm. Letters are used to inform participants of their allocation.

### Learning therapy group (cognitive intervention group)

The cognitive intervention method is the same as that used in our previous study using learning therapy for healthy older adults [30]. Training tasks use two simple tasks (solving arithmetic and Japanese language problems) that are systematized basic problems in arithmetic and reading [30,36]. We prepared various materials used in everyday classes of first-grade to fourth-grade elementary school students. The problems are printed on both sides of an A4 sheet of paper (210 × 297 mm). For the arithmetic problems, the lowest level of difficulty is single-digit addition. The highest level is three-digit division. For the Japanese language problems, the lowest level of difficulty is reading and writing simple sentences. The highest level is reading fairy tales aloud.

One or two weeks before the start of the intervention program, intervention group participants are asked to go to a classroom. The appropriate level of difficulty and workload for all participants is assessed by diagnostic tests, which consist of 70 arithmetic and 16 language problems. The arithmetic problems range in difficulty from single-digit addition to three-digit division. The language problems range in difficulty from reading and comprehending Japanese *Haiku *(17 characters) to reading and comprehending stories (110 characters). For tests of both types, the percentage of correct answers and the time it takes to solve all problems are determined. In this intervention, the difficulty level and workload of each task are set such that each participant is able to solve the problems with ease and without mental stress within 15 min.

The cognitive intervention is scheduled to be conducted for 23 weeks. Participants in the cognitive intervention group are asked to go to the classroom in Tohoku University once a week. They are instructed to complete five sheets of each task prepared for each for that day, which are assessed by staff members. Mistakes are corrected by the participants themselves. The study period ends when the participants complete each of the problems correctly. The daily learning time for the two tasks is approximately 15 min. The cognitive intervention is undertaken on an individual basis, so that the participants can decide how to use their learning time of 15 min freely. Participants are also asked to do their homework of two tasks for 4 to 6 days a week. Regarding their homework, participants are asked to complete five sheets of each task prepared for each. Furthermore, participants are asked to bring back their achievements of homework on the next school day. The staff members check their homework and provide advice when necessary.

### Waiting list group (no cognitive intervention group)

The wait-listed group receives no intervention. Those participants are informed by letter that they are scheduled to receive an invitation to participate after a waiting period of 6 months. No placebo is used for the social contact group. Results of previous intervention studies [26,68] report that a placebo group is unnecessary for this type of study because no difference exists in cognitive or functional improvement between the placebo and no-social-contact groups (control group).

### Overview of cognitive function measures

To evaluate the beneficial effects of learning therapy on cognitive functions, we assess a broad range of cognitive functions (Table 1). Measures of the cognitive functions are divisible into seven categories (executive functions, episodic memory, short-term memory, working memory, reading ability, attention, and processing speed). Executive functions are measured using the Stroop test (ST) [69] and verbal fluency task (VFT) [70]. Episodic memory is measured using logical memory (LM) [71] and first and second names (FS-N) [72]. Short-term memory is measured using digit span forward (DS-F) [65]. Working memory is measured using digit span backward (DS-B) [65]. Reading ability is measured using the Japanese reading test (JART) [67]. Attention is measured using the digit cancellation task (D-CAT) [73]. Processing speed is measured using digit symbol coding (Cd) [65] and symbol search (SS) [65]. Details of all tasks are described below.

**Table 1:** Summary of cognitive function measures

Cognitive function	Task
Executive functions	Stroop test

	Verbal fluency task

Episodic memory	Logical memory

	First and second names

Short-term memory	Digit span forward

Working memory	Digit span backward

Reading ability	Japanese reading test

Attention	Digit cancellation task

Processing speed	Digit symbol coding

	Symbol search

We assess these cognitive function measures before and after the intervention period (6 months). The primary outcome measure is ST. We selected ST as the primary outcome measure because: (1) learning therapy is expected to improve executive functions, and a previous study showed learning therapy can improve executive function measured by FAB [30]; (2) ST is a task that is often used to measure executive functions [74,75]; and (3) ST has been standardized, with high reliability and validity in Japanese populations [69,76].

#### ST

Stroop test (ST) measures executive function including response inhibition and impulsivity. Hakoda's version is a paper and pencil version ST [69]. In this test, participants must check whether their chosen answers are correct, unlike the traditional oral naming ST. We use a reverse ST and a ST. In the reverse ST, in the leftmost of six columns, a word naming a color is printed in another color (for example 'red' is printed in blue letters); the other five columns are each filled with five different colors from which participants must check the column whose color matches the written word in the leftmost column. In the ST, in the leftmost of six columns, a word naming a color is printed in another color (for example 'red' is printed in blue letters) and the other five columns contain words naming colours. Participants must check the column containing the word naming the color of the word in the leftmost column. In each task, participants are instructed to complete as many of these exercises as possible in 1 min. The primary measure for this task is the number of correct items.

#### VFT

Verbal fluency task (VFT) measures executive function. We use the Japanese version of VFT [70], which has two tasks (letter fluency task (LFT) and category fluency (CFT) task). In LFT, a Japanese letter, 'ka', is given to each participant, who is then asked to generate common nouns beginning with this letter - as many as possible in 60 s. In CFT, a category name (animal) is given to each participant, who is then asked to generate many words of a certain category (animal). The participants are instructed not to include proper nouns or to repeat one that has already been stated. The primary measure for this task is the number of words reported. The reliability and validity of Japanese LFT were demonstrated by Ito [70].

#### LM

Logical memory (LM) evaluates the performance of episodic memory. LM is a subtest of the Wechsler Memory Scale-Revised (WMS-R) [71]. LM consists of two short paragraph-length stories (Story A and Story B). In LM, participants must memorize the short story. The stories are scored in terms of the number of story units recalled, as specified in the WMS-R scoring protocol. We use either Story A or Story B. The primary measure for this task is the number of correct story units recalled.

#### FSN

First and second names (FSN) evaluates memory ability in everyday life. FSN is a subset of Rivermead Behavioral Memory Test (RBMT) [72]. RBMT measures episodic memory as it is used in everyday life. Therefore, subsets of RBMT are similar to everyday situations. In FSN, participants must memorize first and second names with faces (photograph). Subsequently, they must recall the first and the second names when the face is shown again later. We use four faces (four first names and four second names). The primary measure of this test is the total number of correct answers in both first and second names. The maximum raw score of FSN is 8.

#### DS

Digit span (DS) is a subtest in Wechsler Adult Intelligence Scale-Third Edition (WAIS-III) [65]. DS, which has two subsections (DS-F and DS-B), evaluates short-term memory and working memory. DS-F measures short-term memory by simply requiring participants to repeat numbers. DS-B measures working memory by requiring participants to memorize numbers and repeat the numbers in the inverse order. For DS-F, participants repeat numbers in the same order as they were read aloud by the examiner. For DS-B, participants repeat numbers in the reverse order of that presented aloud by the examiner. In both, the examiner reads a series of number sequences which the examinee must repeat in either forward or reverse order. DS-F has 16 sequences. DS-B has 14 sequences. The primary measures of this test are raw scores that reflect the number of correctly repeated sequences until the discontinue criterion (that is, failure to reproduce two sequences of equal length) is met [65]. The maximum raw score of DS-F is 16. The maximum raw score of DS-B is 14.

#### JART

The Japanese reading test (JART) measures reading ability [67]. JART is a Japanese version of the National Adult Reading Test (NART) which has a reading test of 50 irregularly spelled words in English (for example ache) [77]. JART is a reading test comprising 25 Kanji compound words (e.g. 親父, 煙草). The reading stimuli are printed out randomly for reading. The participants are asked to read each Kanji compound word aloud. This task assesses reading ability and IQ. The primary measure for this task is the number of correct items.

#### D-CAT

Digit cancellation task (D-CAT) evaluates attention [73]. The test sheet consists of 12 rows of 50 digits. Each row contains five sets of numbers 0 to 9 arranged in random order. Consequently, any one digit appears five times in each row with randomly determined neighbours. D-CAT consists of three such sheets. Participants are instructed to search for the target number(s) that had been specified to them and to delete each one with a slash mark as quickly and as accurately as possible until the experimenter sends a stop signal. There are three trials, first with a single target number (6), second with two target numbers (9 and 4), and third with three (8, 3, and 7). Each trial is given for 1 min. Consequently, the total time required for D-CAT is 3 min. In the second and third trials, it is emphasized that all the target numbers instructed should be cancelled without omission. The primary measure of this test is the number of hits (correct answers). We use only the number of hits in the first trial.

#### Cd

Digit symbol coding (Cd) is a subtest of WAIS-III [65]. This test measures processing speed. For Cd, the participants are shown a series of symbols that are paired with numbers. Using a key within a 120 s time limit, participants draw each symbol under its corresponding number. The primary measure of this test is the number of correct answers.

#### SS

Symbol search (SS), a subtest of WAIS-III containing 60 items [65], measures processing speed. For this subtest, participants visually scan two groups of symbols (a target group and a search group) and report whether either of the target symbols matches any symbol in the search group. Participants respond to as many items as possible within a 120 s time limit. The primary measure of this test is the number of correct answers.

### Sample size

Our sample size estimation is based on the change score in the reverse ST, which is the primary outcome in this study. We expected to detect a large effect size (*η*^2 ^= 0.14) of the change score in the reverse ST between learning therapy and waiting list groups. the sample size was determined using G * power [78,79] based on 80% power, a two-sided hypothesis test, an alpha level of 5%, an analysis of covariance (ANCOVA) model that includes a baseline reverse Stroop task score, age, and sex as a covariate. The sample size calculation indicated that we need 32 participants in each of the learning therapy and waiting list groups with consideration of a 20% drop-out rate.

### Analysis

This study is designed to evaluate the beneficial effect of learning therapy in elderly people. We calculate the change score (post-training score minus pre-training score) in all cognitive function measures. We conduct an ANCOVA for the change scores in each cognitive test. The change scores are the dependent variable. Groups (learning therapy, waiting list) are the independent variable. Pre-training scores in the dependent variable, sex, age categories are the covariates to exclude the possibility that any pre-existing difference of measure between groups affect the result of each measure and to adjust for background characteristics. The level of significance is set at p < 0.05. Moreover, we report eta squared (*η^2^*) as an index of effect size. It is the standardized difference in the change score between intervention groups (learning therapy group, waiting list group). In actuality, *η^2 ^*≥ 0.01 is regarded as a small effect, *η*^2 ^≥ .006 as a medium effect, and *η^2 ^*≥ 0.14 as a large effect [80]. Missing data are imputed using the expectation-maximization method, as implemented in the Statistical Package for the Social Sciences (SPSS) Missing Value Analysis. It imputes missing values using maximum likelihood estimation with the observed data in an iterative process [81]. All randomized participants are included in the analyses in line with their allocation, irrespective of how many sessions they complete (intention-to-treat principle). All analyses are performed using SPSS software (ver. 18 or higher).

## Discussion

This study is designed to investigate the beneficial effects of learning therapy on widely various cognitive functions such as executive functions, episodic memory, short-term memory, working memory, reading ability, attention, and processing speed in healthy elderly people.

This study has several strengths compared to earlier studies using cognitive training for elderly people. First, this study was designed according to CONSORT guidelines [66]http://www.consort-statement.org/consort-statement/, which are intended to improve standards of reporting of randomized clinical trials (RCT). Consequently, this study has been structured to enable its reproduction in both research and clinical settings. Moreover, we can provide sufficient evidence of the effectiveness of cognitive training such as learning therapy.

Second, we investigate the beneficial effects of learning therapy on widely various cognitive functions. The measures assess cognitive functions of seven categories: executive functions, episodic memory, short-term memory, working memory, reading ability, attention, and processing speed. Various cognitive functions are necessary to support our actions and behaviours in everyday life. For instance, when we cook meals, we must: (1) choose a menu (executive functions); (2) remember the refrigerator contents (memory); (3) seek and select seasonings from storage (attention); and (4) cut and prepare cooking ingredients with speed and efficiency (processing speed). Because of the complexity of such an apparently simple task, it is expected to be important to investigate the beneficial effects of learning therapy on widely various cognitive functions.

Third, we use simple, easily learned training tasks (reading aloud and simple calculation) using paper and pencil. Most training tasks in previous studies were complex tasks using computers [18,31,82-84]. Using computers might make it easy to record data precisely and to control tasks. Nevertheless, elderly people often have difficulty using computers [85-87]. The difficulty using computers might cause frustration and other negative emotion, possibly reducing their motivation to continue. Our training tasks are more familiar to elderly people and thus expected to encourage their willingness.

This study has some limitations. A first limitation is the intervention period. Our intervention period is about 6 months. Some previous studies have shown that short-term intervention (four example 4 to 6 weeks) improved cognitive function in elderly people [18,20]. Considering reduced costs for elderly people, shorter intervention studies using learning therapy would be also needed. A second limitation is participants. We recruit only healthy elderly people for participation in this study. Providing the validity of effects of learning therapy on widely various cognitive functions, we must conduct the same randomized controlled trial (RCT) for non-healthy elderly people such as those with dementia or depression.

In summary, this study is the first to reveal the beneficial effects of learning therapy on a wide range of cognitive functions in elderly people. Our study is designed to provide sufficient evidence of effectiveness of learning therapy. Given that most cognitive functions decrease with age [1] and that these functions are strongly correlated with daily life activities [7-9], our results can elucidate the effects of cognitive training for elderly people.

## Trial status

Recruitment of participants begins in February 2012, and is expected to end in January 2013.

## Abbreviations

ANCOVA: Analysis of covariance; Cd: Digit symbol coding; CONSORT: Consolidated standards of reporting trials; D-CAT: Digit cancellation task; DS-B: Digit span backward; DS-F: Digit span forward; FS-N: First and second names; IQ: Intelligence quotient; JART: Japanese reading test; LM: Logical memory; MRI: Magnetic resonance imaging; RCT: Randomized controlled trial; RBMT: Rivermead behavioral memory test; SPSS: Statistical package for the social sciences; SS: Symbol search; ST: Stroop test; UMIN: University Hospital Medical Information Network; VBM: Voxel based morphometry; VFT: Verbal fluency task; WAIS-III: Wechsler Adult Intelligence Scale-Third Edition; WMS-R: Wechsler memory scale-revised.

## Competing interests

Learning therapy was developed by RK and KUMON Institute of Education. However, RK derives no income from KUMON Institute of Education and Society for Learning Therapy. RK has no other competing interests. All other authors have declared no competing interests.

## Authors' contributions

RN designed, developed the study protocol, and calculated the sample size. RN and HN searched the literature, selected cognitive function measures, created manuals to conduct and rate cognitive measures, and recruited testers for cognitive function measures. HN conducts cognitive function measures and rates these cognitive function measures with testers. RN supervises testers. RN wrote the manuscript with YT, HT, HH, YN, AS, HN, and RK. RK also gave advice related to the study protocol. All authors read and approved the final manuscript.

## Supplementary Material

Additional file 1 CONSORT 2010 checklist of information to include when reporting a randomized trial*. Click here for file
